# Comparison and Feasibility of Various RFID Authentication Methods Using ECC

**DOI:** 10.3390/s18092902

**Published:** 2018-09-01

**Authors:** Pagán Alexander, Rania Baashirah, Abdelshakour Abuzneid

**Affiliations:** Department of Computer Science and Engineering; University of Bridgeport; Bridgeport, CT 06604, USA; apagan@my.bridgeport.edu (A.P.J.); rbaashir@my.bridgeport.edu (R.B.)

**Keywords:** authentication, RFID, elliptic curve cryptography, Random Access Control, security, privacy, IoT, lightweight protocol

## Abstract

Radio frequency identification (RFID) is a technology that has grown in popularity and in the applications of use. However, there are major issues regarding security and privacy with respect to RFID technology which have caught the interest of many researchers. There are significant challenges which must be overcome to resolve RFID security and privacy issues. One reason is the constraints attached to the provision of security and privacy in RFID systems. Along with meeting the security and privacy needs of RFID technology, solutions must be inexpensive, practical, reliable, scalable, flexible, inter-organizational, and long-lasting. To make RFID identifiers effective and efficient they must identify the item(s) while resisting attacks aimed at obtaining the tag’s information and compromising the system or making it possible to bypass the protection RFID tags are supposed to provide. Different authentication methods have been proposed, researched, and evaluated in the literature. In this work, we proposed our methodology in evaluating RFID authentication, and a few of the most promising authentication methods are reviewed, compared, and ranked in order to arrive at a possible best choice of protocol to use.

## 1. Introduction

RFID wireless technology uses radio frequency signals to communicate between the tags which are attached to objects, and the readers, that identify the objects and are connected to a back-end server. RFID is a technology that has grown in popularity and has found many applications across multiple industries. Most people have seen RFID tags used in their everyday lives but may not have realized what the technology behind them is. Every time we go shopping we see RFID tags, they are on the items we are trying to purchase, from clothing to books to games, and even medicines. Many companies have been using RFID technology to maintain and track their inventory, while others have recently been experimenting with its use for secure entry and access control.

**Figure 1:**
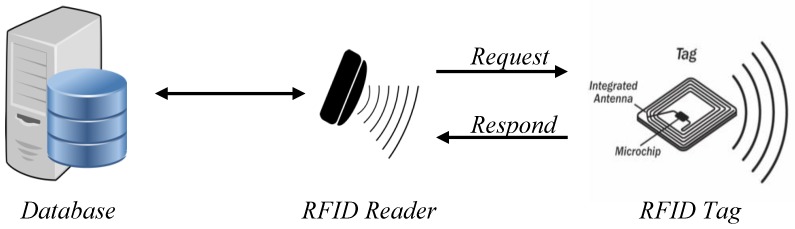
Basic RFID Model.

RFID systems have three main parts, the tag, the reader, and the server, as depicted in Figure 1. The server contains the database housing all of the identification information for the tags and the objects to which they are attached. The reader is the part of the system that plays the middleman between the tags and the server. It reads the tags’ information and confirms it against what is held on the database of the server. The reader and server in the majority of cases have a wired connection which is considered secure. The tag is the most populous part of the system. There will be numerous tags in a system communicating with multiple readers, all running off of one database. The allure of using RFID is that it does not require line of sight in order to communicate between tag and reader, and multiple tags can be read simultaneously.

One use of RFID systems is in loss prevention, where the tags are used to uniquely identify the object they are attached to while providing a measure of security to that object against theft. This makes it a great optional for many Internet of things (IoT) and AI applications. When someone tries to remove the object with an RFID tag from the vicinity, readers near the exit sound an alert if the tag has not been deactivated, only to mention some of the most common applications of RFID.

Another use of the RFID tags has been in stock and inventory control. RFID technology communicates using wireless radio signals, therefore line of sight is not needed for a tag and reader to communicate, unlike barcodes, that require line of sight with a laser reader. Another tremendous advantage over the barcoding is that barcoding requires scanning one item at a time, which is tedious and time-consuming and can also be greatly influenced by human error. RFID tags can simultaneously communicate with a reader that is within range and the reader can read and identify multiple tags simultaneously. As long as each object has been properly tagged, the reader can quickly and easily identify the tagged objects, saving time and lowering the possibility of human error in inventory tracking.

In recent years, the use of RFID technology, specifically in the High Frequency (HF) 13.56 MHz range has received quite a bit of attention. In this range particularly, the NFC standard has been developed and improved. There are five NFC types, each corresponding to different ISO/IEC and JIS standards. Types 1, 2, and 4 are covered under ISO/IEC 14443-A while only Type 4 is covered under both ISO/IEC 14443 A/B. Type 3 NFC is covered by JIS X 6319, and Type 5 by ISO/IEC 15693 (18000-3) [1]. Manufacturers, such as NXP Semiconductor (Newburyport, MA, USA), have pushed NFC forward with their ntag213/215/216 products. Each is Type 2 ISO/IEC 1443 A compliant with memory capacities of 144, 504, and 888 bytes and a data rate of 106 kbps. These NFC cards are ideal for use in access control to improve secure access for enterprises.

However, RFID systems, since they broadcast wirelessly, have downfalls; securing any kind of a wireless system is always a huge concern. Radio signals are omnidirectional and can be picked up by an antenna that can operate on the same frequency. We see this kind of interference all the time. If you have ever listened to the radio and heard walkie-talkie or closed band (CB) radio interference at odd moments, more common nowadays are cell phones near speakers that will produce a static (white noise) type of sound. Even with wireless networks, the signal broadcast goes out in every direction (on most standard antennae). Due to the susceptibility of wireless communications to interference, thus possible security weaknesses, securing RFID communications has become paramount.

In the years of its various industry applications, RFID security has been studied, researched, improved, and the process shall continue. Many authentication protocols for RFID have evolved over the years, but many have failed to accomplish the security goals needed for the application, and some have not only met but surpassed these same security goals. For those that have met the security goals, the biggest question in application comes in the form of cost. What would it cost for this technology to be used? Not that the monetary cost for the system is not a concern, but more so the cost in time of running the system and the cost of the tags needed to secure the system. It would matter more to any organization if the RFID system were affordable, but took a long time to authenticate and identify the tagged objects, in such a case a company can lose customers, due to wait time and incur more costs due to employee productivity, or lack thereof. However, an organization may be willing to pay a little more for an RFID system that is secure and runs quickly enough to not disrupt the natural flow of business. One more concern is the size of tags. The more sophisticated circuit we use the bigger the tag is. The types of RFID tags used will vary based on company needs, if the company is looking to use low frequency (LF) low capacity RFID tags, the use of elliptic curve cryptography protocols may well exceed the available memory capacity on the tags. In those instances, other lightweight protocols that can preserve anonymity while providing secure authentication such as those proposed by Gope in [2,3] may be a more feasible choice. For use with NFC cards, such as the ntag21x’s mentioned above, which include ECC for originality signatures, and can be programmed as needed, exploring multiple ECC authentication protocols for use in secure authentication for access control is a natural approach.

For the purposes of this paper, we focus on public key cryptography, specifically Elliptic Curve Cryptography (ECC). Our goal in this paper is to survey and compare seven different RFID ECC authentication protocols based on their computational costs, communication costs, storage costs, security features, and ability to resist various attacks. A successful RFID protocol must be lightweight, secure, easy to implement, and able to be scaled up or down based on company needs. In all the research for secure communications, many methods have been developed. The seven protocols compared in this paper were selected because they each incorporate randomization, secure authentication, and are lightweight. All of the studied protocols use Elliptic Curve Cryptography to secure the communication between tag and reader, though each takes a slightly different approach. To compare each protocol on the even ground we make the following assumptions:

1. The connection between the reader and server is wired and secure, so the focus is on communication between tag and reader.
2. NIST secp160R1 a 160-bit ECC base is used for each protocol.
3. Scalar multiplication speed, the time needed to perform a 160-bit ECC calculation on a 5 MHz tag is 0.064 s.
4. Any XOR or Scalar addition calculation time is negligible and therefore not factored into cost calculations.
5. Storage rank comparisons are based on 1000 tags.
6. The RFID tags have a memory capacity of 504 bytes.

The testing of each protocol compared in this paper is based on the original authors’ testing methodology and results, which were then taken and applied using the NIST secp160R1 standard and recalculated to compare each protocol on equal terms. With these assumptions in place, the rest of the paper is organized as follows:

- In Section 2, related work on ECC studies is reviewed.
- In Section 3, the various methods studied will be summarized.
- In Section 4, each of the methods will be compared in three cost areas: computational, communication, and storage cost; and two security areas: security features and attack resistance.
- In Section 5, each of the reviewed protocols will be ranked based on the 5 criteria covered in Section 4.
- In Section 6, we discuss the results of the data comparisons and provide an overall rank of the protocols. We will conclude and determine which of the studied methods provide the best-case implementation for RFID authentication.

## 2. Related Work

Many studies have been conducted over the last decade on the use of RFID over standard barcodes. The advantages of using RFID tags in place of barcodes greatly outweighed their shortcomings. However, with the advancement of technology over the last 10 years and its forward progress, the weaknesses inherent in RFID have become more problematic. Some of these weaknesses as stated in [4] are replay attacks, impersonation attacks, brute-force attacks, denial-of-service attacks, man-in-the-middle attacks, and tracking attacks. To address these weaknesses various methods of encryption and authentication have been researched. All the studies researched are conclusive in the use of elliptic curve cryptography (ECC) in providing the most effective base for RFID authentication. Each covers different ECC application methods to meet their goal.

ECC protocols, such as elliptic curve discrete logarithm problem (ECDLP) and elliptic curve factorization problem (ECFP), with random numbers generated to produce keys by both the tag and receiver are investigated in [4,5,6,7,8,9,10,11,12,13,14,15,16,17,18]. These random numbers are included in the calculations to create a key and send it to the receiving device that responds with its own challenge based on the information received to provide mutual authentication. Zheng et al. [5] use an ECC protocol similar to the others but with an elliptic curve Diffie-Hellman (ECDH) key agreement protocol used between the tag and reader. Each study demonstrates that using only one ECC protocol will provide only one-way authentication and leaves the overall system vulnerable. With the addition of a second ECC protocol, two-way authentication has been proven to be established and therefore providing better security to the system overall. The differences in the coupling of the protocols lead to different efficiency and security improvements in the system. For this survey, we will compare the protocols suggested by Alamr et al. [4], Liao et al. [18], Zheng et al. [5], Zhang et al. [13], Zhoa [15], Jin et al. [16], and Dinarvand [17]. While surveying the literature, we believe these articles have provided the top seven methods for RFID authentication using ECC. In addition, we were able to set up a similar test-bed for all the proposed methods so we can have accurate comparisons. Our proposed test-bed could be applied to many other methods as well.

As we compare each protocol we will focus on the aspects of computational costs, storage costs, communication costs (traffic), security features, and attack resistance. The security features we are looking for are mutual authentication, confidentiality, anonymity, availability, scalability, forward security, location privacy, and data integrity. Additionally, we are comparing resistance to the following types of attacks: man-in-the-middle (MIMA), replay, impersonation, key compromise, location tracking, denial-of-service (DoS), cloning, server spoofing, and desynchronization. A comparison of how each of these studied protocols has met the requirements and aspects has been conducted to draw a conclusion as to which ECC protocol combination will be most effective and efficient in providing a low-cost, lightweight, secure RFID system.

## 3. ECC Methods

Each of the protocols uses the same base elements through their calculations, however, authentications vary. The list below describes the common variables and values of each protocol:

P: base point on elliptical curve (320-bits)
S_S_: secret key of the server/reader (160-bits)
S_P_: public key of the server/reader (320-bits)
T_S_: secret key of the tag (160-bits)
T_P_: public key of the tag (320-bits)
A_S_: authentication value of server/reader (320-bits)
A_T_: authentication value of tag (320-bits)
r_1_, r_2_, r_3_: randomly generated numbers (160-bits each)
T: 1000, the sample number of tags in the system for comparison.

**Figure 2:**
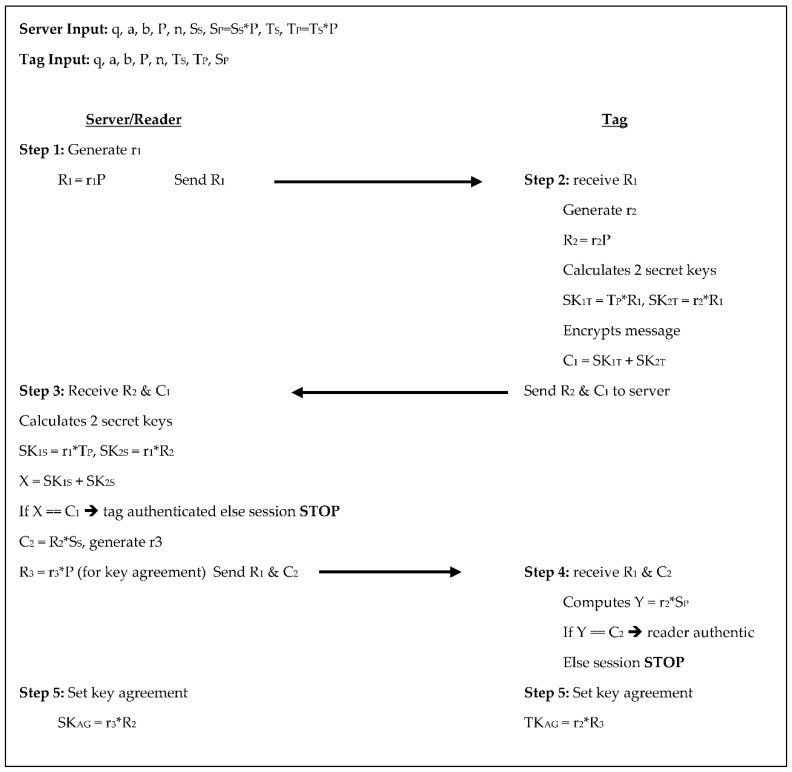
Authentication phases for Alamr et al. [4].

In the Alamr et al. [4] protocol the server chooses a random number for S_S_ and T_S_ and calculates S_P_ and T_P_, respectively. From here, the protocol takes five steps to authenticate between the tag and reader undergoing four scalar multiplications on the tag side and five on the reader/server side, see Figure 2 below. In step 1, the server generates a random number r_1_, then calculates R_1_ = r_1_×P and sends R_1_ to the tag. In step 2, the tag receives R_1_ from the server. The tag then generates a random number r_2_ and calculates R_2_ = r_2_×P. The tag also calculates two secret keys SK_1T_ = T_P_×R_1_ and SK_2T_ = r_2_×R_1_, then encrypts the key as C_1_ = SK_1T_ + SK_2T_ and sends R_2_ and C_1_ to the server. The server in step 3 receives R_2_ and C_1_ then calculates two secret keys of its own, SK_1S_ = r_1_×T_P_ and SK_2S_ = r_1_×R_2_ then calculates X = SK_1S_ + SK_2S_. The server then compares X and C_1_, if they are not equal the tag is not authenticated, and the session stops, otherwise, the server calculates C_2_ = R_2_×S_S_, generates another random number r_3_ and calculates R_3_ = r_3_×P to be used for key agreement. The server then sends C_2_ and R_3_ to the tag. The tag receives C_2_ and R_3_ in step 4 then computes Y = r_2_×S_P_. The tag then compares Y and C_2_, if they are not equal the server is not authenticated, and the session stops, otherwise, the server is authenticated, and both the tag and server set their key agreement in step 5. In step 5 both the tag and the server set their key agreements as TK_AG_ = r_2_×R_3_ and SK_AG_ = r_3_×R_2_. These steps require communicating two scalar products from tag to reader and three scalar products from reader to tag. The tag stores its key pair and the system parameters for a total of 1920-bits, the reader/server stores its key pair, the system parameters, and the T_P_ for each tag. This equates to 1120 + 320T bits.

**Figure 3:**
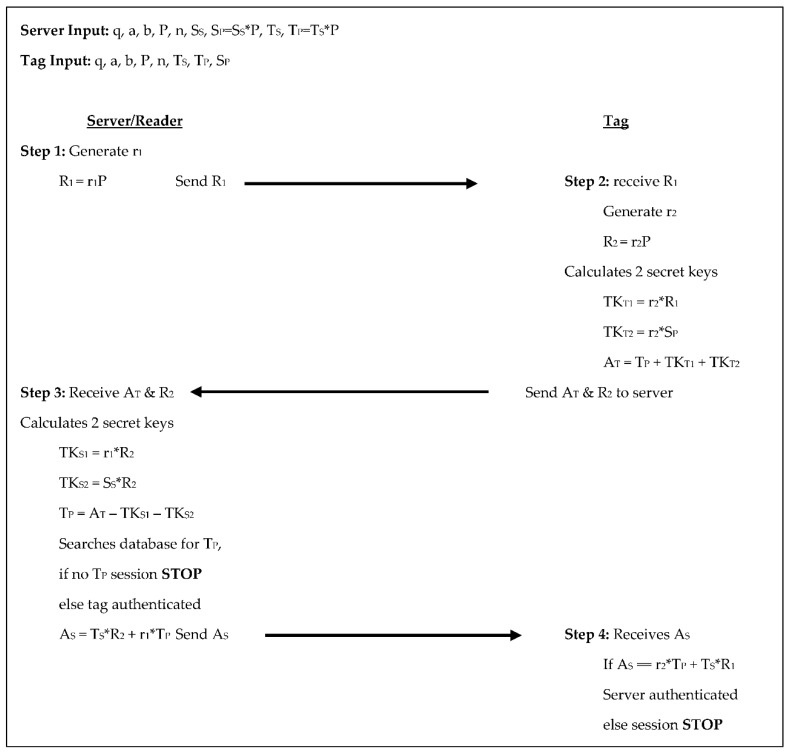
Authentication phases for Liao et al. [18].

The Liao et al. [18] protocol has the server set the domain parameters for both the server/reader and the tag. It chooses random numbers for S_S_ and T_S_ and calculates S_P_ and T_P_ respectively. It has a 4-step authentication process consisting of five scalar multiplications for each the tag and the reader/server, see Figure 3 below. In step 1 the server generates a random number r_1_, calculates R_1_ = r_1_×P, and sends R_1_ to the tag. In step 2 the tag receives R_1_, generates a random number r_2_, calculates R_2_ = r_2_×P, calculates two temporary secret keys: TK_T1_ = r_2_×R_1_ and TK_T2_ = r_2_×S_P_, and sets its authenticator A_T_ = T_P_ + TK_T1_ + Tk_T2_. The tag then sends A_T_ and R_2_ to the server. For step 3 the server receives A_T_ and R_2_ from the tag, computes two temporary secret keys of its own: TK_S1_ = r_1_×R_2_ and TK_S2_ = S_S_×R_2_, and then computes T_P_ as A_T_ − TK_S1_ − TK_S2_. If T_P_ is in the server’s database then the tag is authenticated, otherwise, the session stops. If the tag is authenticated, then the server sets its authenticator A_S_ = T_S_×R_2_ + r_1_×T_P_ and sends A_S_ to the tag. Finally, in step 4 the tag receives A_S_ and compares A_S_ to r_2_×T_P_ + T_S_×R_1_, if they are equal the server is authenticated, otherwise the session stops. For this protocol, there are two scalar products each, communicated from the tag to the reader/server and from the reader/server to the tag. In this protocol the tag stores its key pair, the reader/server’s public key, and the domain parameters totaling 1920-bits, whereas, the reader/server stores its key pair, the system parameters, and the key pair for each tag, equaling 1280 + 800T bits.

**Figure 4:**
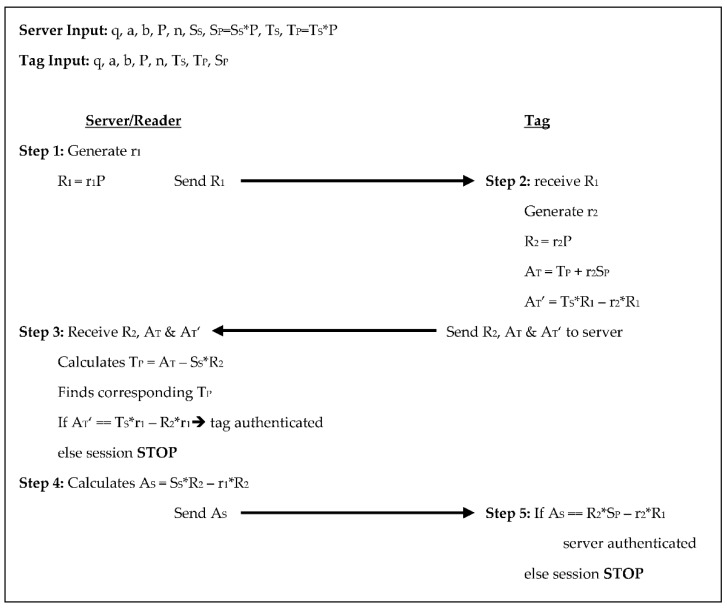
Authentication phases for Zheng et al. [5]

The Zheng et al. [5] protocol includes an ID in addition to the common variables listed above. In this protocol, the server and tag each choose a random number on their own that is assigned as their private keys, the tag’s ID is the same as its T_P_. There is a 5-step authentication process in this protocol with three scalar multiplications for the tag and four for the reader/server, see Figure 4 below. In step 1 the server generates a random number r_1_, calculates R_1_ = r_1_×P, and sends R_1_ to the tag. In step 2 the tag receives R_1_, generates a random number r_2_, and calculates R_2_ = r_2_×P. The tag also computes two authenticators A_T_ = T_P_ + r_2_×S_P_ and A_T_’ = T_S_*R_1_ − r_2_×R_1_, then sends R_2_, A_T_, and A_T_’ to the server. In step 3 the server receives R_2_, A_T_, and A_T_’. The server then calculates T_P_ from A_T_ − S_S_×R_2_ and finds the corresponding T_P_ in its database. Then the server compares A_T_’ to T_S_×r_1_ − R_2_r_1_, if they are equal then the tag is authenticated, and the protocol continues, otherwise, the session stops. In step 4 the server calculates its authenticator A_S_ = S_S_×R_2_ − r_1_*R_2_ and sends A_S_ to the tag. For step 5 the tag receives A_S_ and compares it to r_2_×S_P_ − r_2_×R_1_, if they are equal the server is authenticated, otherwise, the session stops. There are two scalar products each exchanged between the reader/server and the tag in this protocol. The tag stores the system parameters along with the tag’s key pair and ID and the server/reader’s public key totaling 2080-bits, while the reader/server stores the system parameters, the reader/server’s key pair, and the ID for each tag which equates to 1760 + 320T bits.

**Figure 5:**
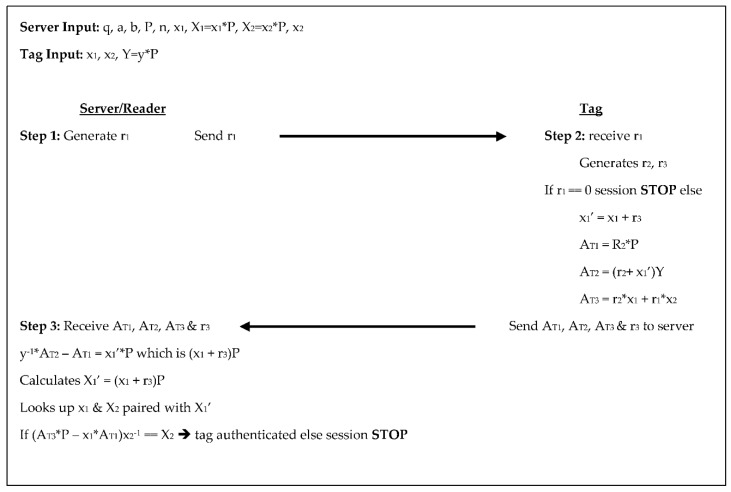
Authentication phases for Zhang et al. [13].

The Zhang et al. [13] protocol was an improvement on ECDLP-based Random Access Control (EC-RAC) proposed by Lee et al. [14]. This protocol, like the original, has two steps for authentication. Both the server/reader and tag generate random numbers, the tag performs four scalar multiplications through the authentication process while the reader/server performs two, see Figure 5 below. In step 1 the server generates a random number r_1_ and sends r_1_ to the tag. In step 2 the tag receives r_1_, generates two random numbers r_2_ and r_3_, then checks r_1_. If r_1_ = 0 then the session stops, otherwise the tag computes X_1_’ = x_1_ + r_3_, where x_1_ and x_2_ are the tags secret keys. The tag also computes three authenticators: A_T1_ = r_2_×P, A_T2_ = (r_2_ + X_1_’) ×Y, and A_T3_ = r_2_x_1_ + r_1_x_2_, then sends A_T1_, A_T2_, A_T3_, and r_3_ to the server. For step 3, the server receives A_T1_, A_T2_, A_T3_, and r_3_ then calculates y^−1^×A_T2_ − A_T1_ = X_1_’×P, remembering that X_1_’×P = (x_1_ + r_3_). The server then searches its database for x_1_ and x_2_ paired with X_1_’ and retrieves the corresponding tag information. Finally, the server compares (A_T3_×P − x_1_*A_T1_) ×x_2_^−1^ to X_2_, if they are equal the tag is authenticated, otherwise the session is stopped. In this protocol, the communication between the reader/server and the tag is a 160-bit randomly-generated-number, while the tag sends back two scalar products and two 160-bit number values. The tag stores the system parameters along with the tag’s key pair, and the reader/server’s public key for a total of 1600-bits, while the reader/server stores the system parameters, its key pair, and the key pair of each tag equating to 1440 + 480T.

**Figure 6:**
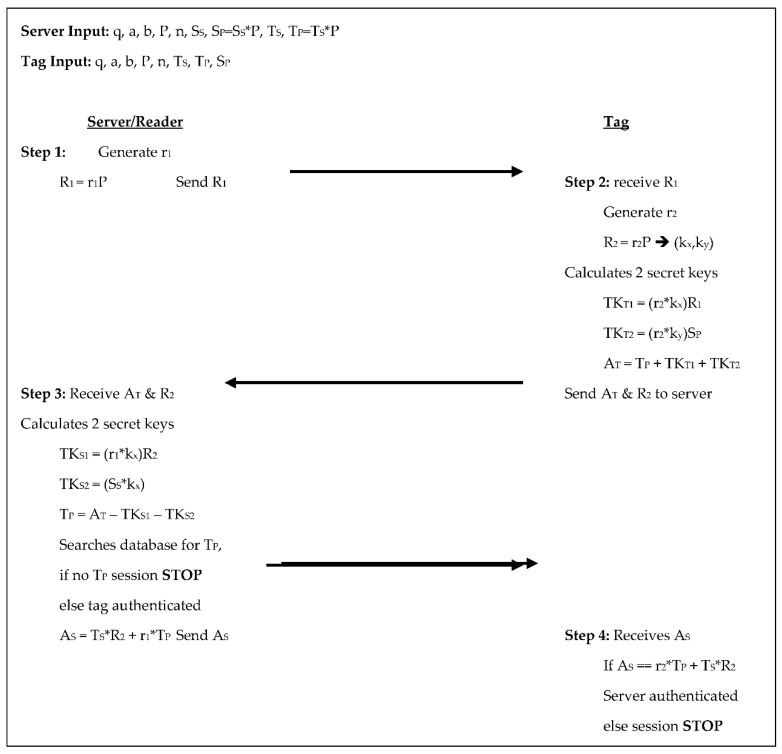
Authentication phases for Zhao [15].

Zhao’s [15] protocol has the reader/server select the domain parameters along with a random number to be used as its private key, while also selecting random numbers for each tag to be used as the tag’s private key; from these the public keys are calculated. Throughout its 4-step authentication process, the tag and reader/server perform five scalar multiplications each, see Figure 6 below. In step 1 the server generates a random number r_1_, calculates R_1_ = r_1_×P, and sends R_1_ to the tag. In step 2 the tag receives R_1_, generates a random number r_2_, and calculates R_2_ = r_2_×P, which it also sees as (k_x_,k_y_). The tag calculates two temporary keys: TK_T1_ = (r_2_×k_x_) ×R_1_ and TK_T2_ = (r_2_×k_y_) ×S_P_, from these the tag’s authenticator is computed as A_T_ = T_P_ + TK_T1_ + TK_T2_, and the tag sends A_T_ and R_2_ to the server. The server receives A_T_ and R_2_ in step 3 then calculates two temporary keys of its own: TK_S1_ = (r_1_×k_x_) ×R_2_ and TK_S2_ = (S_S_×k_y_). The server uses these to determine T_P_ from A_T_ − TK_S1_ − TK_S2_ and searches its database for T_P_. If T_P_ is not in the database the session is stopped, otherwise, the tag is authenticated, the server retrieves the tag’s corresponding information and calculates its authenticator A_S_ = T_S_×R_2_ + r_1_×T_P_ and sends A_S_ to the tag. In step 4 the tag receives A_S_ and compares it to r_2_×T_P_ + T_S_×R_2_, if they are equal, the server is authenticated, otherwise, the session is terminated. The tag communicates two scalar products to the reader/server and the reader/server communicates two scalar products to the tag. The tag in this protocol stores the system parameters, its public and private keys, and the reader/server’s public key totaling 1760-bits. The reader/server stores the system parameters, its own public, and private keys, along with the key pair for each tag, which is 1120 + 480T bits.

**Figure 7:**
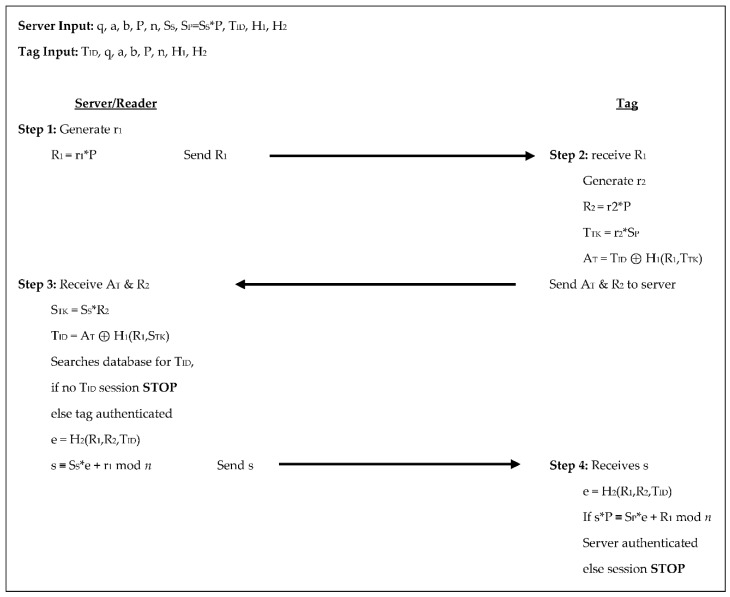
Authentication phases for Jin et al. [16].

In the Jin et al. [16] protocol, like many of the other protocols, the reader/server selects the domain parameters and chooses random numbers to be used as the reader/server’s private key and the private keys for each of the tags. This 4-step authentication process yields four scalar multiplications from the tag and three scalar multiplications from the reader/server, see Figure 7 below. In step 1 the server generates a random number r_1_, calculates R_1_ = r_1_×P, and sends R_1_ to the tag. In step 2 the tag receives R_1_, generates a random number r_2_, calculates R_2_ = r_2_×P, sets a temporary key T_TK_ = r_2_×S_P_, and uses these values with a predetermined hash function, H_1_, to calculate its authenticator, A_T_ = T_ID_ ⊕ H_1_(R_1_,T_TK_) and sends A_T_ and R_2_ to the server.

The server receives A_T_ and R_2_ in step 3, sets its temporary key as S_TK_ = S_S_×R_2_, then compares T_ID_ to A_T_ ⊕ H_1_(R_1_,S_TK_). If T_ID_ is not in the server’s database the session is terminated, otherwise, the tag is authenticated and the server uses another predetermined hash function, H_2_, to calculate e = H_2_(R_1_,R_2_,T_ID_) and s ≡ S_S_×e + r_1_ mod n. The server then sends s to the tag. In the 4th and final step the tag receives s from the server, calculates e = H_2_(R_1_,R_2_,T_ID_) and checks if s×P ≡ S_P_×e + R_1_ mod n if they are not the session is stopped, otherwise, the server is authenticated. In this protocol, the tag sends two scalar products to the reader/server throughout the authentication process and the reader/server sends two scalar products to the tag. The tags store the domain parameters along with its key pair and the reader/server’s public key for 1600-bits of storage; the reader/server stores the domain parameters, its key pair, and the identifier for each tag leading to 1120 + 320T bits of storage.

**Figure 8:**
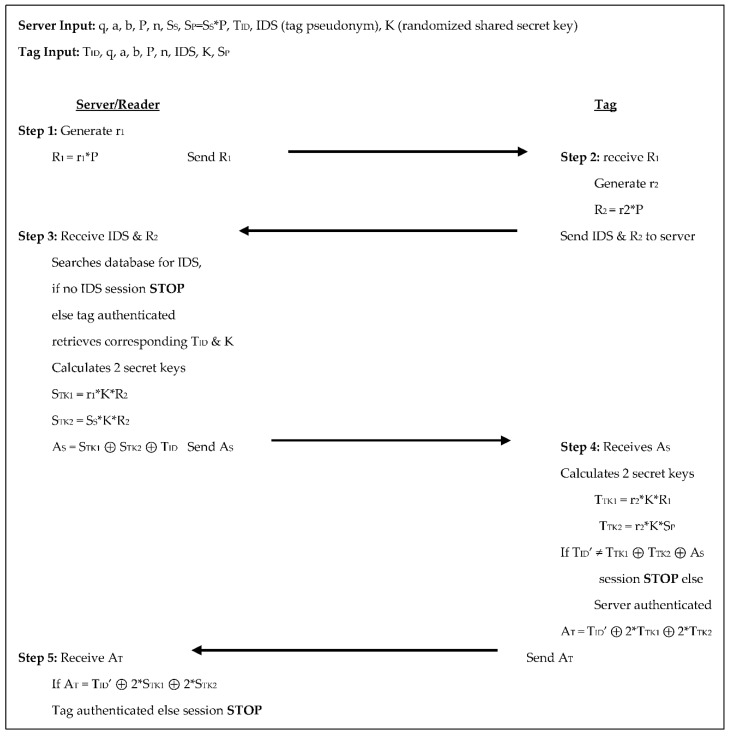
Authentication phases for Dinarvand et al. [17].

The Dinarvand et al. [17] protocol has a more complex approach. The reader/server selects the domain parameters and a random number for the reader/server’s private key. It also selects random points on the elliptic for each tag to serve as the tag’s unique ID, it also selects two random numbers for each tag to serve as the tag’s pseudonym and the shared secret key between the tag and reader/server respectively. Throughout the five authentication steps, there are three scalar multiplications for each the tag and reader/server, see Figure 8 below.

In step 1 the server generates a random number r_1_, calculates R_1_ = r_1_×P, and sends R_1_ to the tag. In step 2 the tag receives R_1_, generates a random number r_2_, calculates R_2_ = r_2_×P, then sends R_2_ and its IDS (tag pseudonym) to the server. In step 3 the server receives R_2_ and IDS from the tag and searches its database for the IDS. If the IDS is not in its database the session is terminated, otherwise, the server retrieves the corresponding shared secret key, K, and tag ID, T_ID_. The server then calculates two temporary secret keys: S_TK1_ = r_1_×K×R_2_ and S_TK2_ = S_S_×K×R_2_. These are used to set its authenticator A_S_ = S_TK1_ ⊕ S_TK2_ ⊕ T_ID_ and then send A_S_ to the tag. In step 4 the tag receives A_S_, calculates two temporary secret keys: T_TK1_ = r_2_×K×R_1_ and T_TK2_ = r_2_×K×S_P_. The tag then compares T_ID_’ to T_TK1_ ⊕ T_TK2_ ⊕ A_S_. If they are not equal the session is stopped, otherwise, the server is authenticated. The tag then calculates its authenticator, A_T_ = T_ID_’ ⊕ 2×T_TK1_ ⊕ 2×T_TK2_ and sends A_T_ to the server. In the 5th and final step, the server receives A_T_ and compares it to T_ID_ ⊕ 2×S_TK1_ ⊕ 2×S_TK2_. If they are not equal the session is terminated, otherwise, the tag is authenticated. The tag sends two scalar products to the reader/server; the reader/server sends two scalar products and a randomly generated 160-bit number to the tag in this protocol. The tag stores the system parameters, its unique ID, the reader/server’s public key, and their shared secret key for 1760-bits of storage. The reader/server stores the system parameters, its key pair, each tag’s unique ID, pseudonym, and shared secret key totaling 1120 + 800T bits of storage.

## 4. ECC Comparisons

In this section, we provide various comparisons between each of the seven protocols. Table 1 shows the number of scalar multiplications for the tag and reader for each protocol and calculates the total computation time based on the aforementioned assumption of 64 ms per scalar multiplication.

Table 2 shows the total communication cost of the tag and reader based on the sizes of the messages exchanged in the protocol in bits. For example, if the tag sends a scalar product and a number to the reader, the message size would be 320-bits for the scalar product plus 160-bits for the number, so the communication cost for that message would be 480-bits. Each protocol summarized above has different message sizes compared in Table 2.

In addition to computation and communication, storage is another factor for comparing the various protocols. If the protocol requires too much storage on either the tag or reader then it would not be scalable nor would it be feasible for use. Table 3 below shows the total storage cost for the tag based on the parameters that are stored on each tag for each protocol, also the storage required for the reader/server is also shown. On the reader/server an additional term, T, is shown; T represents the number of tags in the system, for the purposes of this comparison T = 1000, to provide an example of the possible number of tags that a system may have.

Security features are vital to any authentication protocol. Table 4 shows each protocol and which security features are provided by the protocol. The security features were verified based on the original authors’ proofs and comparisons made in some of the other researched studies. Only 3 of the 7 protocols provide all the listed security features, Liao et al. [18], Zheng et al. [5], and Dinarvand et al. [17].

In addition to the security features, the protocol must be able to resist multiple types of attacks so that the system will not be compromised. The attack resistances were verified based on the original authors’ proofs and comparisons made in the other researched studies. Table 5 shows each protocol and which types of attack they can resist. In this table a yes means that the protocol can resist that specified attack type.

Based on the comparisons made in Table 1, Table 2, Table 3, Table 4 and Table 5 the next section ranks each of the protocols; there are rankings for each protocol based on costs and security.

## 5. ECC Rankings

All of the rankings in this section look at the tables covered in Section 4 and sort orders the protocols based on their computational, communication, and storage costs on both the tag and the reader/server. Additionally, the ranking has been done based on the number of security features met and the number of attacks the protocol can resist.

Table 6 and Table 7 below show the total computational time for each protocol and rank them from lowest computational time to highest; Table 6 shows the rankings for the tag while Table 7 shows the rankings for the reader/server computational costs.

Communication is also a very important protocol factor if the messages take too long to be exchanged that can lead to availability delays which are more hurtful than helpful to the system. Table 8 and Table 9 below rank each protocol based on the communication cost of the messages sent by both the tag and the reader. The cost is in bits, for example, a protocol whose tag sends 2 scalar products to the reader/server would have a message size of 320-bits times 2 for a total message size of 640-bits, based on the 160-bit base for the ECC.

Storage space is another concern for each protocol. The protocol must be able to accomplish its goals of low computational and communication cost while maintaining a reasonable amount of required storage space on the already capacity limited tags. Each protocol stores the system parameters on both the tag and reader/server, what varies are the additional parameters that are stored on the tag and server. With storage, the concern is more on the tag than the reader/server as the reader/server’s memory can be upgraded, that option does not exist with a tag. Table 10 and Table 11 show the storage costs in bits for both the tag and reader/server. Note that the reader/server shows an additional term, T, which represents the number of tags in the system. Those terms with a T, refer to what the reader/server stores for each tag. All amounts are in bits and T was set to 1000 for purposes of numerical comparison and ranking.

Security features such as mutual authentication, confidentiality, data integrity, and so on, are very important when deciding which protocol to use. Table 12 ranks each protocol based on the total number of security features the protocol meets from Table 4. As shown, only three protocols meet all eight features.

Finally, to go along with the provided security features, the protocol must also be able to resist multiple types of attacks. Based on Table 5, each protocol was ranked according to the number of attacks it can resist.

## 6. Data Discussion and Conclusion

With all of the cost comparisons: computational, communication, and storage, coupled with the comparisons of the security features and attack resistances, the rankings were produced. However, individual category rankings do not allow us to determine which protocol would perform best. To make this determination, an average rank value was calculated from each protocol’s rank in Table 6, Table 7, Table 8, Table 9, Table 10, Table 11, Table 12 and Table 13. Based on this average rank value, the protocols were resorted and are listed in rank order in Table 14. Keep in mind that all ranks were handled equally and no category rank was weighted differently. Due to this, the highest-ranking protocol turns out to be Jin et al. [16], which despite having the best average rank score, has what I consider to be a major failing, it does not provide data integrity. The next two protocols on the list do provide all of the security features in addition to attack resistance.

## Figures and Tables

**Table 1:** Comparison of scalar multiplication costs in each protocol.

Elliptic Scalar Multiplication Costs					
Protocol	Tag	Reader	Calculation Speed on 5 MHz Tag (ms)	Total Tag Calculation Time (ms)	Total Reader Calculation Time (ms)
Alamr et al. [4]	4	5	64	256	320
Liao et al. [18]	5	5	64	320	320
Zheng et al. [5]	3	4	64	192	256
Zhang et al. [13]	4	2	64	256	128
Zhao [15]	5	5	64	320	320
Jin et al. [16]	4	3	64	256	192
Dinarvand et al. [17]	3	3	64	192	192

**Table 2:** Cost of communications between tag and reader.

Communication Cost (bits)		
Protocol	Tag	Reader
Alamr et al. [4]	640	960
Liao et al. [18]	640	640
Zheng et al. [5]	640	640
Zhang et al. [13]	960	160
Zhao [15]	640	640
Jin et al. [16]	640	640
Dinarvand et al. [17]	800	640

**Table 3:** Comparison of storage needed in tag and reader/server.

Parameter Storage Cost (bits)		
Protocol	Tag	Reader
Alamr et al. [4]	1920	1120 + 320T
Liao et al. [18]	1920	1280 + 800T
Zheng et al. [5]	2080	1760 + 320T
Zhang et al. [13]	1600	1440 + 480T
Zhao [15]	1760	1120 + 480T
Jin et al. [16]	1600	1120 + 320T
Dinarvand et al. [17]	1760	1120 + 800T

**Table 4:** Comparison of security features met by the various protocols.

Security Features Comparison							
Feature	Alamr et al. [4]	Liao et al. [18]	Zheng et al. [5]	Zhang et al. [13]	Zhao [15]	Jin et al. [16]	Dinarvand et al. [17]
Mutual Authentication	Y	Y	Y	N	Y	Y	Y
Confidentiality	Y	Y	Y	Y	Y	Y	Y
Anonymity	Y	Y	Y	Y	Y	Y	Y
Availability	N	Y	Y	Y	Y	Y	Y
Scalability	N	Y	Y	N	Y	Y	Y
Forward Security	Y	Y	Y	Y	Y	Y	Y
Location Privacy	Y	Y	Y	Y	Y	Y	Y
Data Integrity	N	Y	Y	Y	N	N	Y

**Table 5:** Comparison of each protocol’s resistance to various attacks.

Attack Resistance Comparison							
Feature	Alamr et al. [4]	Liao et al. [18]	Zheng et al. [5]	Zhang et al. [13]	Zhao [15]	Jin et al. [16]	Dinarvand et al. [17]
MIMA	Y	Y	Y	Y	Y	Y	Y
Replay	Y	Y	Y	Y	Y	Y	Y
Impersonation	Y	N	Y	Y	Y	Y	Y
Key Compromise	Y	N	Y	Y	Y	Y	Y
Location Tracking	Y	Y	Y	Y	Y	Y	Y
DoS	N	Y	Y	N	Y	Y	Y
Cloning	Y	Y	Y	Y	Y	Y	Y
Server Spoofing	Y	Y	Y	N	Y	Y	Y
Desynchronization	N	Y	Y	NA	Y	Y	Y

**Table 6:** Ranking based on tag computational cost.

Computational Ranking		
Protocol	Total Tag Calculation Time (ms)	Rank Order
Zheng et al. [5]	192	1
Dinarvand et al. [17]	192	1
Alamr et al. [4]	256	2
Zhang et al. [13]	256	2
Jin et al. [16]	256	2
Liao et al. [18]	320	3
Zhao [15]	320	3

**Table 7:** Ranking based on reader/server computational cost.

Computational Ranking		
Protocol	Total Tag Calculation Time (ms)	Rank Order
Zhang et al. [13]	128	1
Jin et al. [16]	192	2
Dinarvand et al. [17]	192	2
Zheng et al. [5]	256	3
Alamr et al. [4]	320	4
Liao et al. [18]	320	4
Zhao [15]	320	4

**Table 8:** Ranking based on tag to reader/server communication cost.

Communication Ranking		
Protocol	Tag	Rank Order
Alamr et al. [4]	640	1
Liao et al. [18]	640	1
Zheng et al. [5]	640	1
Zhao [15]	640	1
Jin et al. [16]	640	1
Dinarvand et al. [17]	800	2
Zhang et al. [13]	960	3

**Table 9:** Ranking based on reader/server to tag communication cost.

Communication Ranking		
Protocol	Reader	Rank Order
Zhang et al. [13]	160	1
Liao et al. [18]	640	2
Zheng et al. [5]	640	2
Zhao [15]	640	2
Jin et al. [16]	640	2
Dinarvand et al. [17]	640	2
Alamr et al. [4]	960	3

**Table 10:** Ranking based on tag storage of required protocol parameters.

Storage Ranking		
Protocol	Tag	Rank Order
Zhang et al. [13]	1600	1
Jin et al. [16]	1600	1
Zhao [15]	1760	2
Dinarvand et al. [17]	1760	2
Alamr et al. [4]	1920	3
Liao et al. [18]	1920	3
Zheng et al. [5]	2080	4

**Table 11:** Ranking based on reader/server storage of required protocol parameters.

Storage Ranking		
Protocol	Tag	Rank Order
Alamr et al. [4]	1120 + 320T	1
Jin et al. [16]	1120 + 320T	1
Zheng et al. [5]	1760 + 320T	2
Zhao [15]	1120 + 480T	3
Zhang et al. [13]	1440 + 480T	4
Dinarvand et al. [17]	1120 + 800T	5
Liao et al. [18]	1280 + 800T	6

**Table 12:** Ranking based on the number of security features each protocol provides.

Security Features Ranking		
Protocol	Number of Features Met	Rank Order
Liao et al. [18]	8	1
Zheng et al. [5]	8	1
Dinarvand et al. [17]	8	1
Zhao [15]	7	2
Jin et al. [16]	7	2
Zhang et al. [13]	6	3
Alamr et al. [4]	5	4

**Table 13:** Ranking based on the number of different types of attacks each protocol can resist.

Attack Resistance Ranking		
Protocol	Number of Attacks Able to Resist	Rank Order
Zheng et al. [5]	9	1
Zhao [15]	9	1
Jin et al. [16]	9	1
Dinarvand et al. [17]	9	1
Alamr et al. [4]	7	2
Liao et al. [18]	7	2
Zhang et al. [13]	6	3

**Table 14:** Sorted rank of each protocol based on the average of all their rankings.

Overall Protocol Rank	
Protocol	Average Rank
Jin et al. [16]	1.5
Zheng et al. [5]	1.875
Dinarvand et al. [17]	2
Zhang et al. [13]	2.25
Zhao [15]	2.25
Alamr et al. [4]	2.5
Liao et al. [18]	2.75
